# Identification of Structural Variants in Two Novel Genomes of Maize Inbred Lines Possibly Related to Glyphosate Tolerance

**DOI:** 10.3390/plants9040523

**Published:** 2020-04-18

**Authors:** Medhat Mahmoud, Joanna Gracz-Bernaciak, Marek Żywicki, Wojciech Karłowski, Tomasz Twardowski, Agata Tyczewska

**Affiliations:** 1 Human Genome Sequencing Center, Department of Molecular and Human Genetics, Baylor College of Medicine, Houston, TX 77030, USA; helmy.medhat@gmail.com; 2 Institute of Bioorganic Chemistry, Polish Academy of Sciences, Noskowskiego 12/14, 61-704 Poznań, Poland; j.gracz@gmail.com (J.G.-B.); twardows@ibch.poznan.pl (T.T.); 3 Department of Computational Biology, Institute of Molecular Biology and Biotechnology, Faculty of Biology, Adam Mickiewicz University in Poznań, Umultowska 89, 61-614 Poznań, Poland; marek.zywicki@amu.edu.pl (M.Ż.); Wojciech.Karlowski@amu.edu.pl (W.K.)

**Keywords:** maize, glyphosate, herbicide resistance, stress responses, genome sequencing, large structural variants, SNPs, indels

## Abstract

To study genetic variations between genomes of plants that are naturally tolerant and sensitive to glyphosate, we used two *Zea mays* L. lines traditionally bred in Poland. To overcome the complexity of the maize genome, two sequencing technologies were employed: Illumina and Single Molecule Real-Time (SMRT) PacBio. Eleven thousand structural variants, 4 million SNPs and approximately 800 thousand indels differentiating the two genomes were identified. Detailed analyses allowed to identify 20 variations within the EPSPS gene, but all of them were predicted to have moderate or unknown effects on gene expression. Other genes of the shikimate pathway encoding bifunctional 3-dehydroquinate dehydratase/shikimate dehydrogenase and chorismate synthase were altered by variants predicted to have a high impact on gene expression. Additionally, high-impact variants located within the genes involved in the active transport of glyphosate through the cell membrane encoding phosphate transporters as well as multidrug and toxic compound extrusion have been identified.

## 1. Introduction

Maize (*Zea mays* L.) originates from Mexico and therefore has adapted to a climate with high temperatures and high light intensity during the day and moderate temperatures at night [1]. Because of its high productivity, importance for food and feed production, and numerous industrial applications, maize has become one of the most important crop species worldwide; it is grown over a wide range of latitudes [2,3]. As a thermophilic plant, when grown in nonoptimal conditions, maize encounters environmental stresses and hence it is crucial to minimize the competition for water, light, and minerals [4] that occurs between maize seedlings and weeds growing in the field. The easiest way to eradicate weeds is the application of herbicides. One of the most popular and widely used (in the cultivation of maize and crops in general) herbicides of high economic value and effectiveness against a wide range of weeds is the nonselective foliar-applied Roundup® [5,6]. Glyphosate, the active ingredient in Roundup^®^, has its primary effect via the inhibition of 5-enolpyruvylshikimate-3-phosphate synthase (EPSPS; EC 2.5.1.19) [7], an enzyme involved in one of the reactions in the shikimate pathway, which leads to the synthesis of aromatic amino acids—tryptophan, phenylalanine, and tyrosine. Exposure to glyphosate causes deficiencies in those amino acids and leads to the consequent inhibition of protein synthesis. Another important aftereffect is the disturbance of carbon flow related to the accumulation of useless shikimate, as well as the drainage of carbon into other essential pathways such as auxin, flavonoid, or quinone biosynthesis [8,9,10,11,12,13,14]. In the past decade, new proteomic [15] and transcriptomic [16,17,18] and metabolomic [19] methods have been applied to analyze glyphosate influence on plant metabolism. Despite these reports, we are still missing a holistic image of plant responses to glyphosate and, more generally, herbicide stress conditions.

*Zea mays* has a large genome of roughly 2.4 gigabases and approximately 40,000 genes, spread across 10 chromosomes (https://plants.ensembl.org/Zea_mays/Info/Annotation). Some of the chromosomes contain highly repetitive heterochromatic domains called “chromosomal knobs”. The genome is transposon-rich and is composed of up to 85% repetitive sequences, such as transposable elements (TEs), and thus, its organization is highly complex [20,21]. Moreover, maize is one of the crop species with high genetic variation between different lines of the same species. These differences might range from 25% to 84% due to the disparities in TE content since the activation and/or loss of TEs has a great impact on genome structure and gene expression. Moreover, single nucleotide polymorphisms (SNPs), small insertions and deletions (indels), and structural variants (SVs) such as copy number variations (CNVs), inversions and translocations also take part in generating substantial differences between maize genomes [22,23]. Numerous studies have shown that genome structure variations are associated with a wide range of plant phenotypic traits, resulting in metabolic fluctuations and different regulation of gene expression [24,25,26,27].

The first fully sequenced maize genome was B73, published in 2009 [21]. In the following years, full or partial genomic sequences of other varieties, such as Palomero Toluqueno, B104, CML247, DK105, EP1, F7, Mo17, PE0075, PH207, W22, and PI 566673, have been released, and the details are available in the MaizeGDB database (https://www.maizegdb.org/genome/assemblies_overview) [28]. Recently, a new version of the maize B73 reference genome was published (RefGen_v4) [22].

In this study, we analyzed the genome structures of two naturally inbred maize lines (S245 and S7957), which differ in resistance to glyphosate. To identify SVs, SNPs and indels which could be involved in gaining herbicide resistance in non-GMO maize, two different sequencing technologies have been applied (Illumina and SMRT PacBio).

## 2. Results

### 2.1. Verification of Sequencing Data

For the purpose of sequencing and to overcome the complexity of the two maize genomes two sequencing technologies: Illumina (two paired-end libraries with insert length of 400 bp and 500 bp generating short but highly accurate sequencing reads) and SMRT PacBio (two mate-pair libraries with insert length of 8 kb and 11 kb, longer but less precise reads) were used. To evaluate the obtained sequencing results, alignment rates and genome coverages for each library were calculated, based on maize B73 genome [21]. For Illumina libraries, the average coverage was high (>67×). Moreover, high alignment rates indicated that both lines are close to the reference genome of the maize B73 line. The examination of the cumulative distribution of genome coverage indicates that approximately 45% of the genome was covered with at least 50 Illumina reads (details are presented in Table 1). For PacBio reads, after correction with the HALC program, the average coverage for both lines was > 3×, with a slightly higher value observed in the sensitive line (3.59×, vs 3.06× in the tolerant line). Due to the low overall coverage of PacBio reads, the saturation of genic and intragenic regions of both genomes was compared. In genic regions, the coverage in the sensitive line was higher than the coverage in the tolerant line (accordingly, 78% vs 62%). Nevertheless, the minimum coverage of 2, which is necessary to identify the structural variants, was observed for the majority of genic and intergenic regions in both lines (55% and 70% of genic regions, 60% and 65% of intergenic regions of tolerant and sensitive lines, respectively). We concluded that both obtained data sets (Illumina and PacBio) will allow the identification of structural variants between the lines.

### 2.2. Identification of Large Structural Variants

**Figure 1:**
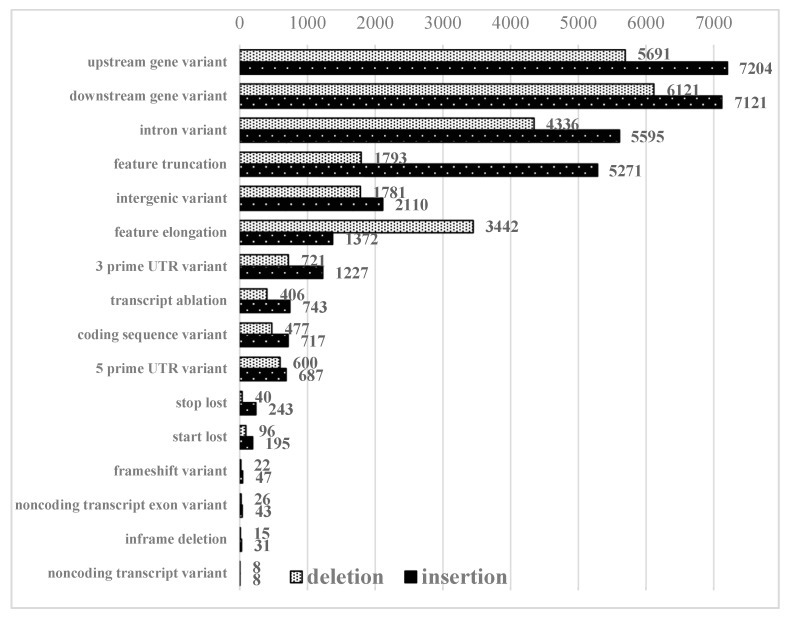
Predicted consequences of identified structural variants.

Long PacBio reads, which are able to cover substantial chromosomal regions and directly reveal large deletions, insertions, duplications, or inversions [29], allowed for detection of structural differences between genomes of selected maize inbred lines. A total of 11,172 structural variants were identified representing 6062 insertions and 5110 deletions (only variants supported with 5 or more reads were investigated). Most variants were located in noncoding regions, such as upstream/downstream regions of the genes and intronic and intergenic regions. These sets of identified SVs were functionally annotated using the Ensembl variant effect predictor (VEP) to verify the probable influence of the identified changes on gene expression levels (Figure 1).

When analyzing such a narrow set of variants, the most frequent effect was transcript ablation (33.2%). Other predicted consequences of identified structural changes, such as coding sequence variation (12.32%), intron variant (9.25%), feature truncation (9.25%), or start/stop codon loss (8.41% and 8.18%, respectively) were less frequent. At the same time, only a few cases of transcript elongation or frameshift were identified. The vast majority of the variants were found to affect noncoding parts of the genome (upstream/downstream regions of the genes (22.16% and 22.75%, respectively), intronic (17.07%), and intergenic regions (6.69%)). Coding sequence variants, transcript ablation, frameshift variants and inframe deletions represented 2.05%, 1.97%, 12%, and 0.08%, respectively. Only those SVs with high possibility impact on phenotype were selected for further analysis.

### 2.3. Identification of Small Structural Changes

For the identification of smaller structural changes, such as SNPs and indels, in genomes of tolerant and sensitive maize lines, we used Illumina reads. After each dataset read was aligned to the reference genome of the maize B73 line, only changes differentiating between both lines were reported. In this way, we obtained a large number of variances (Table 2).

Considering that maize line B73 is sensitive to glyphosate [30] for a more accurate prediction, the pool of SNPs and indels was narrowed to those occurring only in the tolerant line. As a result, a comprehensive list of genetic variations observed exclusively in glyphosate-tolerant line has been obtained. This approach resulted in the identification of 4,068,829 SNPs and 729,866 indels (Table 2), among which 113,775 SNPs and 15,277 indels were located within the protein coding regions.

**Figure 2:**
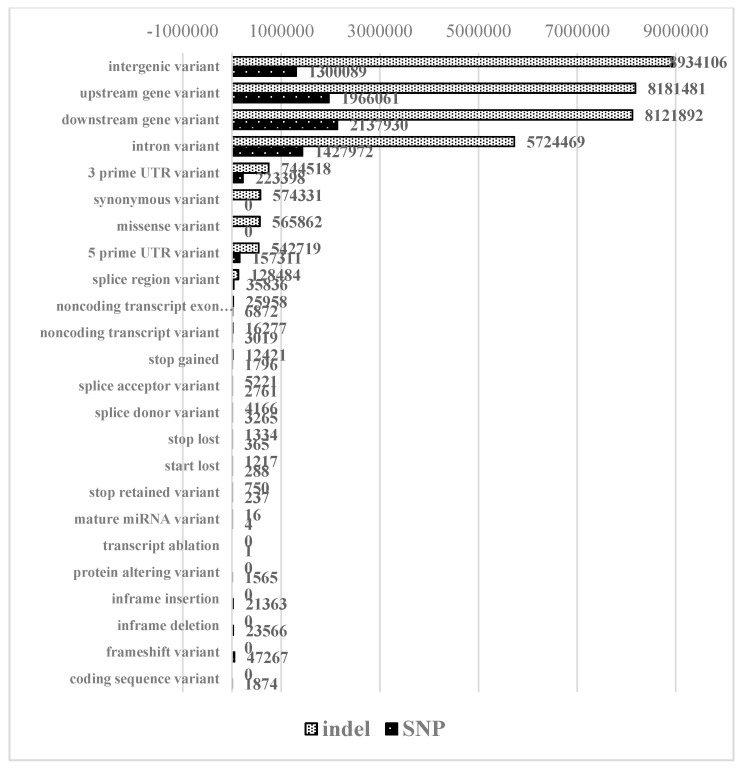
Predicted high impact consequences of identified indels and single nucleotide polymorphisms (SNPs).

To gain the insight into functional consequences of the identified variants, the annotation with Ensembl variant effect predictor (VEP) has been performed. The results for all and high impact consequences are given in Figure 2. The vast majority of the variants were found to affect noncoding (intergenic regions (25.13%), upstream/downstream regions of the genes (24.9% and 25.18%, respectively), and intronic (17.56%) parts of the genome.

### 2.4. Gene Ontology Analysis

Analysis of the distribution of gene ontology terms associated with genes influenced by high-impact large structural variants was performed in the next step. The obtained list of GO terms was summarized using REViGO. Among the genes affected by deletions, the most abundant GO terms in molecular function division were iron ion binding, methyltransferase activity, transferase activity and RNA binding, while in the biological process category, the most abundant terms were glycogen biosynthesis, protein K48-linked deubiquitination, carbohydrate metabolism and metal ion transport. It is worth noting that some of those genes were also annotated as a part of the response reaction to heat, nematode and oxidative stress such as Zm00001d037385, which encodes the adenine nucleotide alpha hydrolase-like superfamily protein or genes Zm00001d052001, Zm00001d010838 (ZIP zinc/iron transport family protein), and Zm00001d002426 (thylakoid lumenal 29 kDa protein, chloroplastic) [31,32,33]. On the other hand, among genes affected by insertions, the most abundant GO terms in molecular function were RNA binding, SUMO transferase activity and hydrolase activity. In the biological process category, protein sumoylation, cell growth, ATP hydrolysis and cellular response to extracellular stimulus were the most common GO terms. In the last category, two MYB transcription factors, Zm00001d029963 and Zm00001d037836, were identified.

Analysis of GO terms associated with high-impact SNPs and indels showed that a group of genes belonging to the RNA binding category was the most affected by those changes, similar to the results from the annotation of large insertions identified by PBSuite. The second most abundant GO term was methyltransferase activity. This group, in turn, was the most abundant GO term within genes affected by large deletions. Notably, in GO terms related to biological processes, the largest group of genes affected by SNPs and indels belonged to the category of DNA repair. These results suggest the existence of potential differences in the ability to maintain genome homeostasis in maize lines tolerant to glyphosate.

## 3. Discussion

The development and maturation of organisms is constantly influenced by environmental conditions that sometimes may exert a detrimental influence on them. Plants, due to their sedentary lifestyle, require short-term strategies to quickly and efficiently readapt their metabolism and have developed unique features in terms of habitat, growth and reproduction, often being a result of differences in their genomes [34].

Genetic variation, being the result of continuous changes ranging from single-nucleotide polymorphisms (SNPs), through indels, to large structural variants (SVs) like chromosomal rearrangements, provides raw material on which both natural selection and human act. Despite the advances in sequencing technologies, SVs continue to be largely unexplored elements of plant genomes. Understanding the type and size of SVs, their distribution among individuals and in population is critical for understanding the contributions of SVs to phenotypes, and the plants’ ability to adapt to changing environments [25].

Since the basic challenge in genome studies is correlating genomic DNA variation with observed heritable phenotypes for our studies, we chose two maize inbred lines that differ in herbicide tolerance (Supplementary Figure S1), a variation that has been observed in the field (see Materials and methods). The differences recognized in both analyzed genomes and their functional diversity suggest a large genetic distance between tested maize inbred lines and that the vast majority of structural variants (SNPs and indels) are related to line-specific variability rather than to the glyphosate tolerance trait. Among all SNPs (13,778,463) and indels (2,443,262) detected in our study 4,068,829 (29.53%) SNPs and 729,866 (29.87%) indels were tolerant-line specific (Table 2) suggesting possible connection with glyphosate tolerance. Most highly represented changes caused by structural variants (for variants with a predicted high impact on gene expression) were transcript ablation (1149 events), coding sequence variant (426), followed by intron variation (320), feature truncation (320), stop loss (283), and 3′ UTR variant (283) (Figure 1).

**Figure 3:**
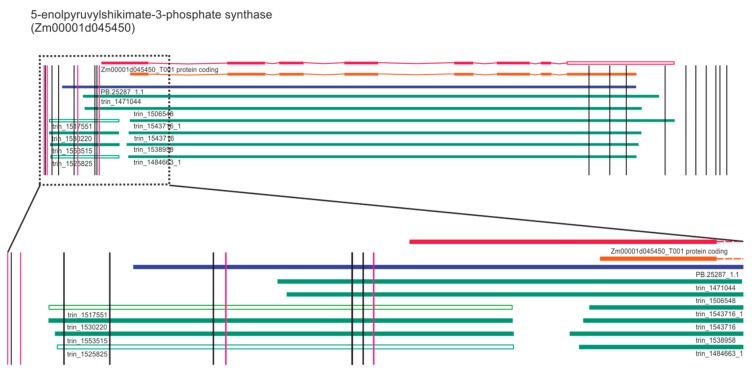
Location of identified SNPs (black) and indels (violet) on the 5-enolpyruvylshikimate-3-phosphate synthase (EPSPS) gene.

To recognize genome changes that are potentially responsible for the tolerant phenotype, we examined genes and pathways involved in glyphosate metabolism like the shikimate pathway (Supplementary Figure S2). First, we analyzed variants of the 5-enolpyruvylshikimate-3-phosphate synthase (EPSPS) gene as the best-known example of an enzyme inhibited by glyphosate [35,36,37,38]. To date several means of overcoming susceptibility to glyphosate have been described; for instance, amino acid substitution in the enzyme’s active site [39,40,41], copy number variation of the gene and its overexpression [42]. In the case of the tolerant maize line studied herein no large structural variants, only several SNPs and indels within or in the vicinity of the EPSPS gene have been identified (Figure 3).

In red, the EPSP gene structure according to the version 4 annotation of the maize genome, and in orange, the version 3 annotation have been given. Blue bars represent the publicly available full-length EPSPS transcript isoform identified with the iso-seq approach; green bars represent publicly available Trinity-assembled transcripts from RNA-seq data. The first part of the genes and transcripts, where the highest number of changes occurs, has been enlarged (indicated by black dashed line) to clearly see the SNPs and indels.

Importantly, all identified changes had, according to VEP, a moderate or modifier impact on gene expression, and none of them were located within the coding region. Previously it has been shown that a mutation in EPSPS coding sequence can provide resistance to glyphosate, as demonstrated in goosegrass, *Eleusine indica* and Pantoea sp. [35,43,44].

**Figure 4:**
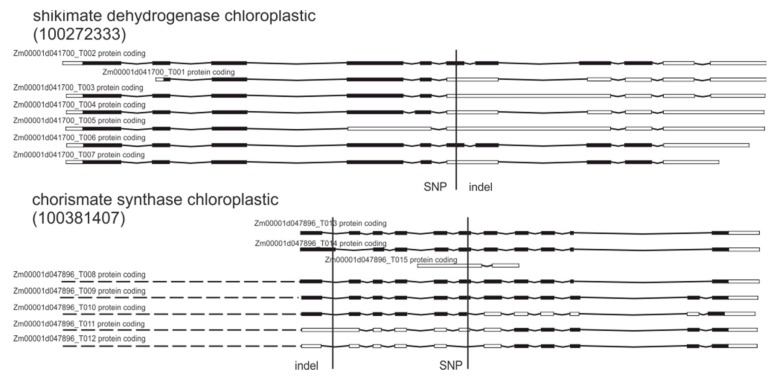
Genetic variations in genes encoding proteins involved in shikimate pathway.

Two other genes coding enzymes involved in the shikimate pathway (bifunctional 3-dehydroquinate dehydratase/shikimate dehydrogenase and chorismate synthase) [45] were also affected by detected variations (Table 3, Figure 4).

For both tested genes (shikimate dehydrogenase chloroplastic and chorismate synthase chloroplastic) the impact on gene expression was high, affecting splice sites and resulting in frame shifts. If these changes lead to higher activity of both enzymes, it would be possible to at least partially compensate for the inhibitory effect of glyphosate on EPSPS by increasing EPSPS substrate synthesis and elevated intake of the EPSPS product.

**Figure 5:**
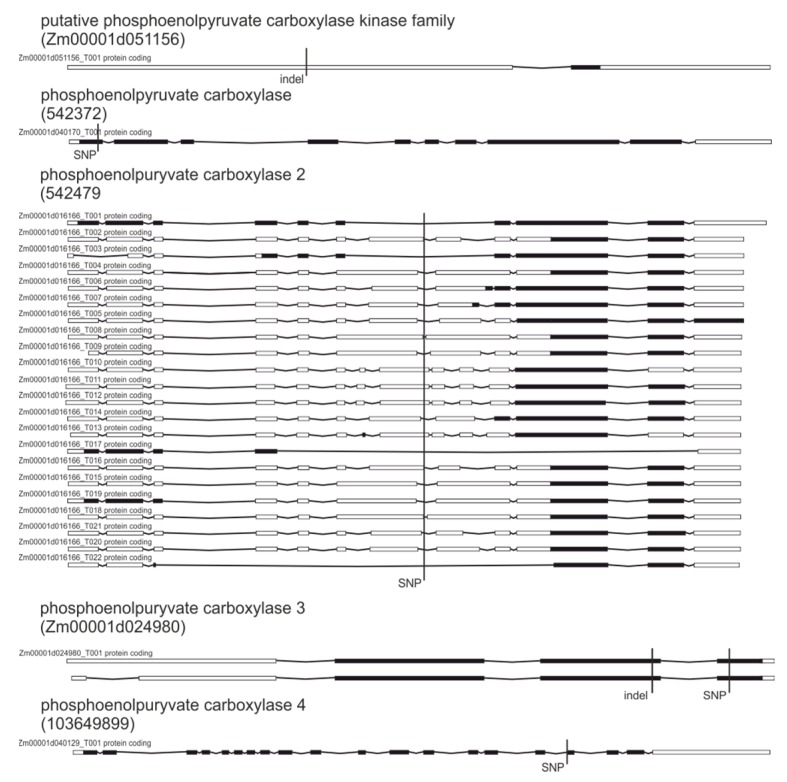
Genetic variations in genes encoding proteins related to lowering the availability of phosphoenolpyruvate for shikimate pathway.

Other identified structural variations that could result in increased tolerance to glyphosate were located in genes encoding phosphate transporters (Table 4, Figure 5).

Previously it has been proposed that phosphate transporters 1 and 2 could participate in the active transport of glyphosate into plant cells [5,8,10,11,14,46]. Other identified transporters with SNP and indel changes (Table 4) participate in the distribution of inorganic phosphate, 3-phosphoglycerate, triose phosphates and, to a lesser extent, phosphoenolpyruvate (PEP), which is a substrate for the shikimate pathway that is directly affected by glyphosate inhibition of the EPSPS gene [47,48].

Ten other genes affected by high-impact changes that are involved in PEP cellular availability are given in Table 5. Proteins encoded by those genes are important for maintaining appropriate concentrations of PEP in chloroplasts, where the shikimate pathway takes place [49,50,51].

**Figure 6:**
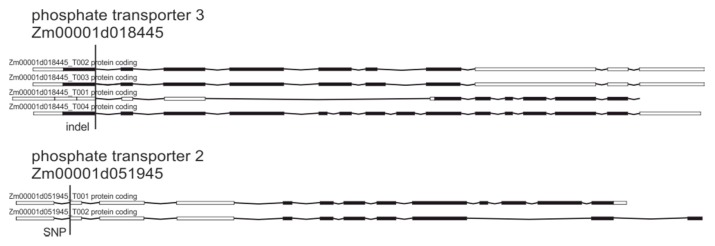
Genetic variations in phosphate transporter genes.

The identified variations could potentially lead to stimulation of the shikimate pathway and at least partial compensation for herbicide action as observed changes cause splice acceptor changes, frame shifts, stop codon loss or gain (Figure 6).

In rice for instance, silencing of the PEPC gene lead to a 50%–60% increase in the activity of the shikimate pathway due to higher PEP availability [52]. On the other hand, increase of shikimic and protocatechuic (PCA) acids in nodules and leaves of nodulated lupine plants after herbicide application were explained by a diversion of most PEP into the shikimate pathway, depriving energy substrates to bacteroids to maintain nitrogen fixation [53].

The next group of genes with high-impact structural variants that drew our attention in the context of herbicide stress responses were genes encoding proteins involved in multidrug and toxic compound extrusion (MATE, summarized in Table 6). This protein family is one of the most conservative and largest transporter families in plants and acts as membrane carriers of drugs and synthetic compounds, as well as organic acids, plant hormones and secondary metabolites [54,55,56].

**Figure 7:**
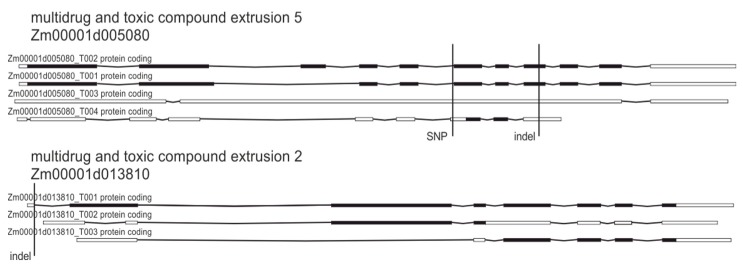
Genetic variations in selected genes encoding proteins involved in multidrug and toxic compound extrusion.

The MATEs gene family of cation antiporters also plays a large role in the exportation of toxins and other substrates and reportedly localize in plasma membranes [56,57]. Identified variants (that cause splice acceptor/donor change, frame shifts, stop codon loss or gain) may lead to the enhancement of glyphosate active transport and lowering of its cellular concentration (Figure 7).

## 4. Conclusions

Harnessing the potential of genetic variations present within plant genomes and especially crop varieties, possible now due to technological and algorithmic advances, will undoubtedly forward our insight in some biological processes and may help in the improvement of agronomic crop species. In the face of increasing demands imposed by the impact of climate change and by a growing world population such improvement becomes necessity nowadays.

The presented results from the sequencing of two maize inbred lines give us insight into the molecular pathways that may be potentially involved in the glyphosate tolerance trait. The identified variations and changes are strong indicators for further biochemical analyses, especially those related to genes encoding three shikimate pathway enzymes (bifunctional 3-dehydroquinate dehydratase/shikimate dehydrogenase and chorismate synthase). The described changes caused by genetic variations can lead to an increase in shikimate pathway efficiency and thus compensate for the decreased activity of the EPSPS caused by glyphosate. Additionally, genes encoding phosphoenolpyruvate carboxylase and its regulator, phosphoenolpyruvate carboxylase kinase, were also affected by identified variants of high impact on gene expression. The potential decrease in phosphoenolpyruvate carboxylase activity could lead to the increased availability of PEP, providing another level of EPSP inhibition compensation. Other mechanisms responsible for tolerance traits could be related to altered glyphosate transport via phosphate transporters or multidrug and toxic compound extrusion proteins. These mechanisms could modify the effective intracellular concentration of glyphosate and allow plants to thrive after Roundup^®^ spraying. All identified differences in sequenced genomes shed a light on new potential ways that maize could respond to herbicidal stress conditions.

## 5. Materials and Methods

### 5.1. Plant Material and Sequencing

Maize seeds from inbred lines S245 (line tolerant to glyphosate, TL) and S79757 (line sensitive to glyphosate, SL) were grown in a greenhouse. Seeds from both lines were obtained from a local breeder (HR Smolice, Smolice, Poland). Field tests were used to verify the responses of the S245 and S7957 lines to glyphosate. When the highest concentration of herbicide (Round Up 360 SL) was used (300 g, 1.0 l/ha) the level of injuries to the tolerant maize variety was 40%, in contrast it reached 85% in the more sensitive line (K. Adamczewski, data not published) (Supplementary Figure S1).

Uniform seedlings from both lines were selected and grown at 22 °C, humidity 60% in the dark room. Seedlings were harvested after 7 days and immediately frozen in liquid nitrogen and stored at −80 °C until shipment. DNA isolation (done from a single seedling for each line), and sequencing were performed as outsourced services by the Fasteris company (Switzerland). Two sequencing technologies were used: Illumina libraries were prepared as two paired-end libraries with insert lengths of 400 bp and 500 bp and two mate-pair libraries with insert lengths of 8 kb and 11 kb; for long reads, Single Molecule Real-Time (SMRT) PacBio, RS II technology was used.

### 5.2. Preparation and Assessment of Sequencing Data

After removing adaptor sequences and sorting, Illumina reads were aligned using BWA-mem with default parameters and setting –M flag to mark split reads as secondary aligned. Coverage was calculated using the plotCoverage script from the deepTools package (https://github.com/fidelram/deepTools). For SMRT PacBio, 38 SMRT cells for the tolerant line and 40 SMRT cells for the sensitive line were sequenced. Reads were aligned using BWA [58] version 0.7.10 using -x PacBio with the following parameters: minimum seed length (-k17), where matches shorter than 17 are removed, max gap set to 40 where gaps longer than 40 are not found (-W40), (-r10) a key heuristic parameter for tuning the performance reseeding for an MEM longer than minimum seed length, matching score 2 (-A2), mismatch penalty 5 (-B5), gap opening penalty 2 (-O2), (-E1) setting gap penalty to gap opening + minimum seed length * E), and clipping penalty 0 (-L0). After the assessment of correction tool performance [59], the HALC program was used for PacBio reads.

### 5.3. Calling Variance

GATK haplotypecaller (GATK HC) version 3.5 [60] was used to call variance between maize lines. The GATK best practice workflow was followed. Variants were filtered using hard filtering for SNPs, with suggested parameters, where SNPs matching any conditions will be marked FILTER and will not be considered in further analysis, and the remaining SNPs will be annotated as PASS (QD < 2.0).

### 5.4. Structure Variation Detection

Two methods of SV identification were employed: Sniffles [61] version 1.0.3 and PBSuite [62] version 15.8.24. In the first step, structural variants were detected with 2 minimum supporting reads, and the threshold was set on at least 5 reads. The comparison between variants detected with multiple approaches was performed using the BEDTools suite. The number of exact overlaps between corrected and uncorrected subreads identified SVs using the BEDTools intersect by setting -f 1.0 for 100% overlap and set–r flag, which requires the fraction of overlap to be reciprocal for both samples.

### 5.5. Functional Analysis

Only SNPs and SVs specific to the Roundup^®^ tolerant line (S245) were further analyzed as potentially responsible for the tolerant phenotype. Changes in the S245 line were filtered out using SnpSift (http://snpeff.sourceforge.net/SnpSift.html), and then Variant Effect Predictor (VEP) was used to annotate selected SNPs. In the case of SVs, the gene ontology term accession associated with the SV affected genes was extracted using plant BioMart from Ensembl Plants genome browser (http://plants.ensembl.org/index.html) and then summarized using REViGO [63].

### 5.6. Sequence Deposition

All sequences have been deposited in ENA (European Nucleotide Archive) database under the following accession number PRJEB31400.

## Figures and Tables

**Table 1:** The alignment results for Illumina libraries in glyphosate-tolerant and glyphosate-sensitive *Zea mays* lines.

	Tolerant Line (S245)			Sensitive Line (S79757)		
Library Size	Millions of Reads	Genome Coverage	Alignment Rate	Millions of Reads	Genome Coverage	Alignment Rate
400 bp	816	37.5	97.1%	821	37.7	96.6%
500 bp	664	27.8	85.3%	615	29.1	97.0%
8 kb	129	3.7	86.4%	74	3.0	87.7%
11 kb	59	2.2	86.0%	116	2.6	70.0%

**Table 2:** Summary of identified small variants from Illumina reads.

Variant Group	SNPs	Indels
All	13,778,463	2,443,262
Tolerant line-specific	4,068,829	729,866
Located in coding sequence	113,775	15,277

**Table 3:** Shikimate pathway genes affected by SNPs and indels.

Gene	Protein	Variation	Consequence
100272333	Bifunctional 3-dehydroquinate dehydratase/shikimate dehydrogenase chloroplastic	SNPs, indels	Splice donor/frame shift
100381407	Chorismate synthase chloroplastic	SNPs, indels	Splice donor/frame shift

**Table 4:** Genes encoding phosphate transporters that were affected by identified variations.

Gene	Protein	Variation	Consequence
Zm00001d051945	Phosphate transporter 2	SNP	Splice acceptor
Zm00001d018445	Phosphate transporter 3	indel	Splice donor
Zm00001d012747	Putative sugar phosphate/phosphate translocator	SNP, indel	Splice donor/Splice acceptor
Zm00001d021653	Glucose-6-phosphate/phosphate translocator 2	indel	Frame shift
100191756	Probable sugar phosphate/phosphate translocator	indel	Frame shift
Zm00001d011388	Putative sugar phosphate/phosphate translocator	indel	Frame shift

**Table 5:** Genes associated with phosphoenolpyruvate availability affected by genetic variations between tested lines.

Gene	Protein	Variation	Consequence
100283648	Phosphoenolpyruvate/phosphate translocator 1 chloroplastic	SNP	Splice acceptor
103649694	Phosphoenolpyruvate/phosphate translocator 2 chloroplastic	indels	Frame shift
Zm00001d044715	Phosphoenolpyruvate/phosphate translocator 2 chloroplastic	indel	Frame shift
Zm00001d037659	Phosphoenolpyruvate/phosphate translocator 2 chloroplastic	SV insertion	Stop lost
542372	Phosphoenolpyruvate carboxylase	SNP	Stop gained
Zm00001d053453	Phosphoenolpyruvate carboxylase isoform 1	SNPs, indels	Splice acceptor/Splice donor
542479	Phosphoenolpyruvate carboxylase 2	SNP	Splice donor
Zm00001d024980	Phosphoenolpyruvate carboxylase 3	SNPs, indels	Stop gained/frame shift
103649899	Phosphoenolpyruvate carboxylase 4	SNP	Splice acceptor
Zm00001d051156	Putative phosphoenolpyruvate carboxylase kinase family protein	SNPs, indel	Stop gained/frame shift

**Table 6:** Multidrug and toxic compound extrusion genes affected by genetic variations.

Gene	Protein	Variation	Consequence
Zm00001d005080	Multidrug and toxic compound extrusion5	SNPs, indels	Splice acceptor/frame shift
Zm00001d013810	Multidrug and toxic compound extrusion2	indels, structural variant (SV) insertion	Frame shift/ start loss
100383875	Multidrug and toxic compound extrusion3	indel	Frame shift
100193278	Multidrug and toxic compound extrusion4	indel	Frame shift
Zm00001d035115	Multidrug and toxic compound extrusion1	indel	Stop gained

## Supplementary Materials

The following are available online at https://www.mdpi.com/2223-7747/9/4/523/s1.
