# Supplementary figures and images for: Identification of Structural Variants in Two Novel Genomes of Maize Inbred Lines Possibly Related to Glyphosate Tolerance
Source: Plants (Basel). 2020 Apr 18;9(4):523. doi: 10.3390/plants9040523 (PMC7238182; doi:10.3390/plants9040523)

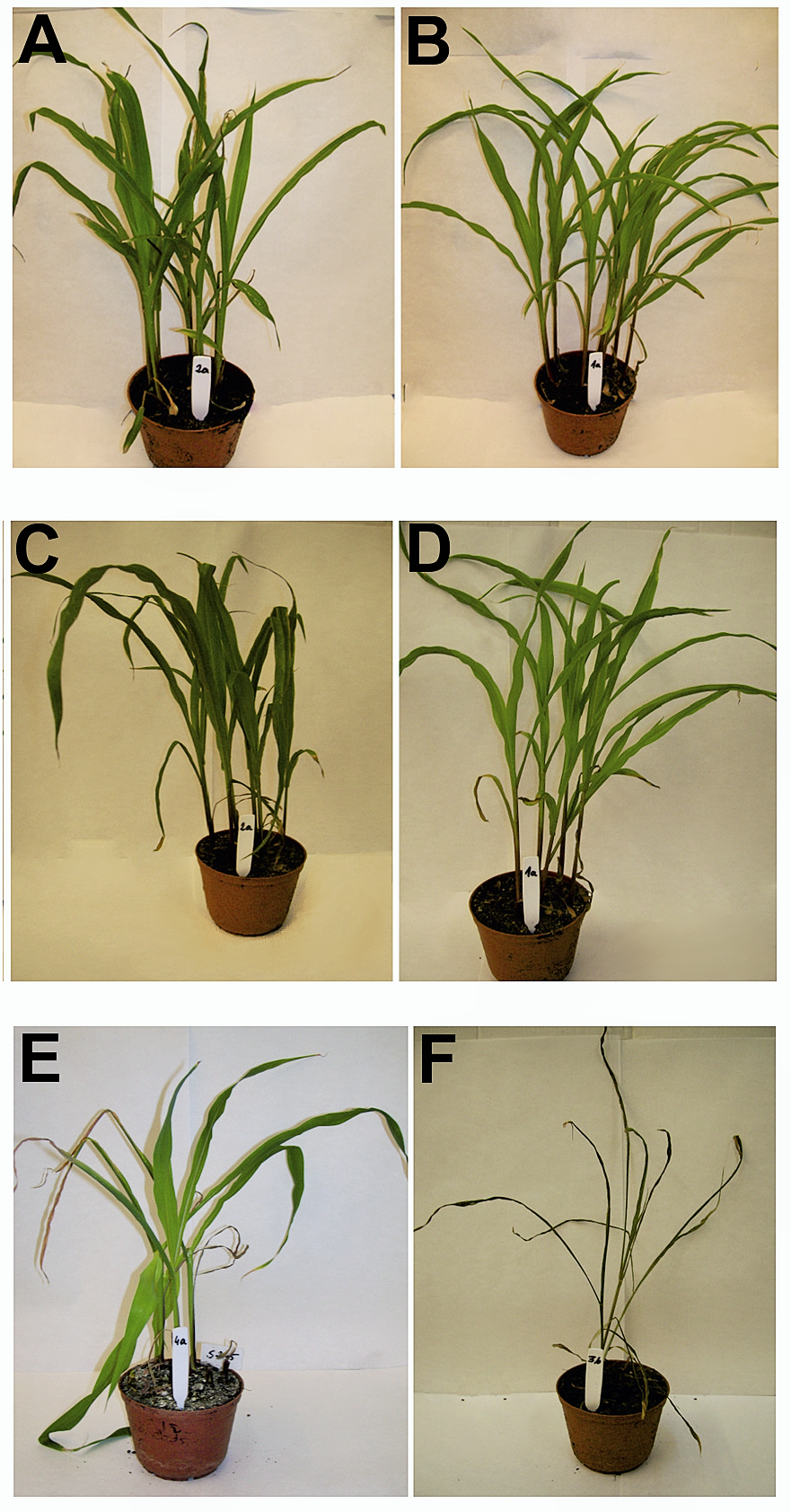

Supplement: Supplementary file 1 [file plants-09-00523-s001.zip › Supplementary Figure 1.png]

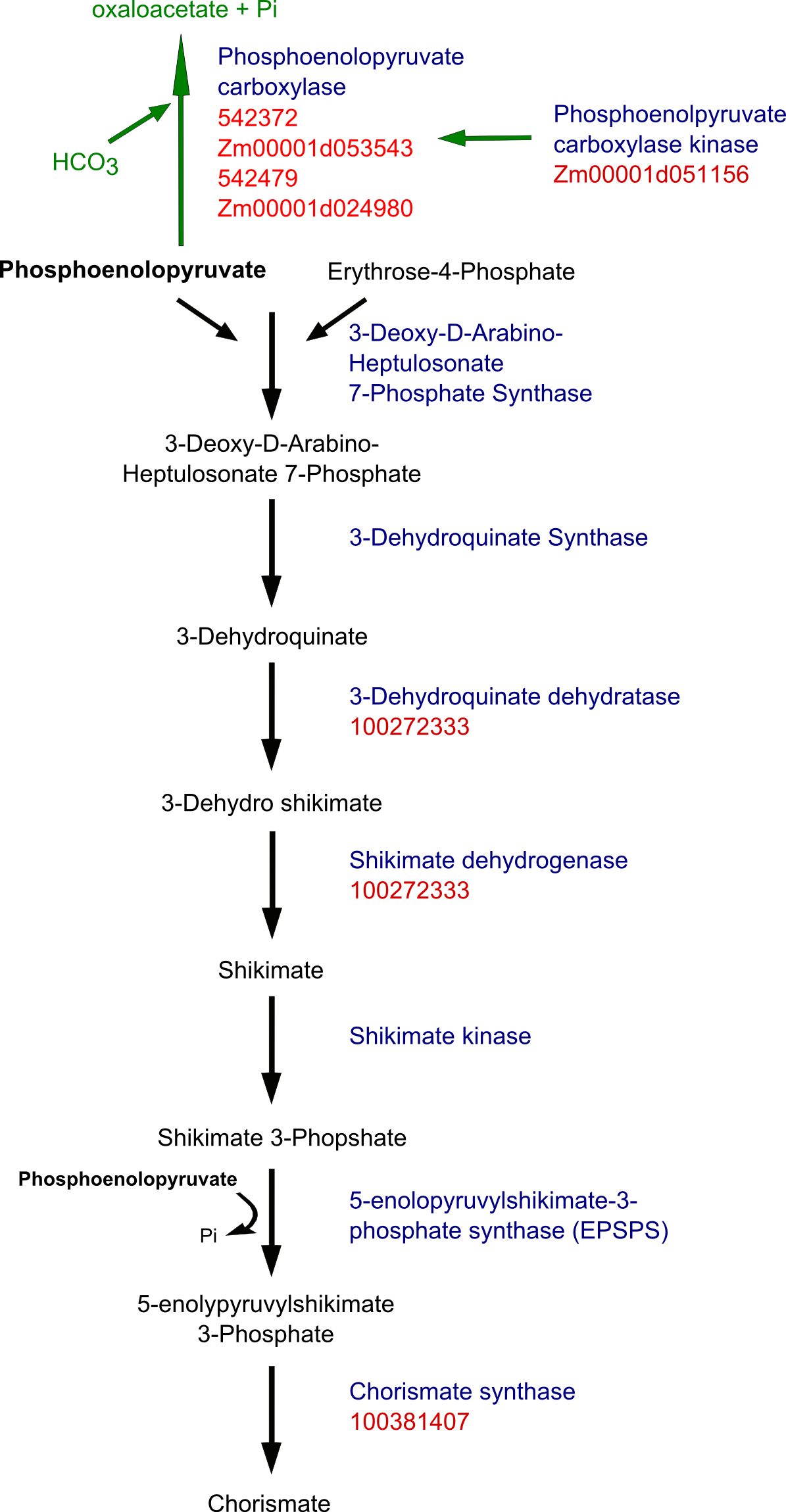

Supplement: Supplementary file 1 [file plants-09-00523-s001.zip › Supplementary Figure 2.png]
